# Kinetics of miR-122 Expression in the Liver during Acute HCV Infection

**DOI:** 10.1371/journal.pone.0076501

**Published:** 2013-10-04

**Authors:** Youkyung Choi, Hans-Peter Dienes, Kris Krawczynski

**Affiliations:** 1 Division of Viral Hepatitis, Centers for Disease Control and Prevention, Atlanta, Georgia, United States of America; 2 Institute of Pathology, University of Cologne, Cologne, Germany; Inserm, U1052, UMR 5286, France

## Abstract

The relationships among micro RNA-122 (miR-122) expression in the liver, hepatitis C virus (HCV) replication and hepatic damage were analyzed in three chimpanzees observed for 180 days after inoculation with HCV genotype 1a. Levels of miR-122 in the liver and serum were measured by real-time RT PCR in serial liver biopsies and serum samples. Hepatic miR-122 levels were normalized separately for each of three chimpanzees with small RNAs and microRNAs that are endogenous to the liver and are stably expressed. Two- to 4-fold rise in hepatic miR-122 levels was observed at the onset of HCV infection (the first 4 weeks) when HCV titers in the liver and serum increased rapidly in all three chimpanzees in concordance with *in vitro* data indicating the miR-122 significance for HCV replication. Between 10 to 14 weeks after inoculation, when hepatic and serum HCV RNA titers exceeded 3 logs and alanine aminotransferase (ALT) activity was elevated, hepatic miR-122 levels were in decline. Cumulative data derived from all three chimpanzees from 180 days of observation documented an inverse (negative) correlation between hepatic miR-122 and HCV RNA in the liver and serum and positive correlation between level of serum miR-122 and HCV replication. Subsequent rise of miR-122 level during HCV clearance and ALT normalization suggested a tri-phasic occurrence of the relationship among hepatic miR-122 expression, HCV replication and hepatic destruction, which was the most apparent in one chimpanzee but less evident in two other animals. *In vivo* kinetics of hepatic and serum miR-122, HCV replication and hepatic destruction reflects complexities of the virus-host interaction during the acute phase of HCV infection.

## Introduction

More than 170 million people world-wide are estimated to be chronically infected with HCV, and in the USA, about 25,000 individuals every year are newly infected by HCV. Viral persistence commonly follows primary infection, which may result in liver fibrosis, cirrhosis, and hepatocellular carcinoma [1], [2]. Among a variety of factors identified to influence HCV replication, mircoRNA (miR)-122 and miR-199 have been reported to be of particular significance [3]–[7]. MicroRNAs are small, noncoding RNA molecules of 19–22 nucleotides in length able to repress gene expression of a broad array of targeted transcripts by either RNA interference or impairment of translational initiation and elongation. MicroRNAs are implicated in a wide variety of cellular processes like cell differentiation, proliferation, and apoptosis [8], [9]. miR-122 is specifically and abundantly expressed in hepatocytes, comprising approximately 70% of total miRNA in the liver [10].

*In vitro* studies showed high expression of miR-122 in Huh7 cells hosting HCV replication and documented interaction of miR-122 with the 5′-untranslated region (UTR) of the HCV RNA genome [4], [11], [12]. Other studies confirmed that miR-122 regulates the abundance and production of infectious HCV [13]. Although miR-122 does not directly stimulate HCV RNA synthesis [14], it facilitates HCV replication by recruiting an RNA-induced silencing complex (RISC) containing Ago2 (Argonaute-2) protein, mediating the stabilization of HCV, and slowing the 5′ decay of the viral genome in infected cells [15]–[17]. Furthermore, it was suggested that miR-122 triggers HCV replication by post-transcriptional repression of heme oxygenase enzyme synthesis [7].

*In vivo* experiments in chronically HCV-infected chimpanzees treated with antisense miR-122-locked nucleic acids revealed significant repression of HCV replication and further implicated miR-122 in virus replication [18]. In view of these findings, blockage of miR-122 has been considered as a therapeutic approach against chronic hepatitis C [19]. During acute and chronic HCV infections, responses from type 1 interferon [interferon alpha (IFN-α) and interferon beta (IFN-β)] produced in the liver are a part of the innate immune response against HCV infection inducing mediators of antiviral responses such as protein kinase K, the 2′ 5′-oligoadenylase synthetase, the adenosine deaminase ADR1, and the Mx GTPase [20]. IFN-β treatment can lead to a significant reduction in the expression of miR-122 levels *in vitro* [11], although no significant correlation has been observed between miR-122 levels and serum HCV RNA titer in chronic hepatitis C patients with IFN-α therapy [21].

Most of the studies outlined above were conducted using *in vitro* models. In this study, hepatic and circulating miR-122 levels were measured serially in chimpanzees undergoing acute HCV infection in order to determine the association between miR-122 expression, HCV replication, and ensuing liver pathology. Critical to the investigation was the development and application of protocols for normalizing hepatic miR-122 expression levels using small RNAs and microRNAs expressed in the liver.

## Materials and Methods

### HCV infection in chimpanzees

All animal procedures were approved by the Institutional Animal Care and Use Committee at CDC and in accordance with the guidelines of the Guide for the Care and use of Laboratory Animals. CDC primate protocol number: 1363KRACHIC. The study was done between Sept.23, 2004 and March 22, 2005. The animals were single or pair housed in accordance with the Guide for the Care and Use of Laboratory Animals in an AAALAC accredited facility. Chimpanzees were housed individually for short periods of time due to the restrictions of the scientific protocol involving the infectious nature of hepatitis research. A 12:12-h light:dark cycle was used at a room temperature of 17.8 to 28.9°C and a relative humidity of 30% to 70%. Water was provided ad libitum through an automatic watering system. The diet consisted of a nonhuman primate chow (Lab Diet High Protein Monkey Diet 5045, PMI Nutrition International, LLC, Saint Luis, MO), fruits, and treats (Bio-serv, Frenchtown, NJ). Environmental enrichment plan for chimpanzees consisted of the following: social enrichment, structural enrichment, manipulanda, novel food items/foraging, and sensory enrichment. Potential suffering during liver biopsy and bleeding procedures was alleviate using anesthetic medication using ketamine mixture and tiletamine HCL/Zolazepam HCL (Telazol). None of chimpanzees included in the study was sacrificed. All chimpanzees (CH6413, CH256 and CH1541) were inoculated intravenously with HCV genotype 1a, varying between 10^3^ to 10^3.5^ chimpanzee infectious doses (CID) [22]–[24]. Serum samples were obtained every week at baseline and during 180 days after inoculation. HCV RNA in sera was tested and quantified by Cobas Amplicor HCV v. 2.0 (Roche Diagnostic Systems, Branchburg, NJ). The VITROS Anti-HCV assay (Ortho-Clinical Diagnostics, Rochester, NJ) was used for detection of anti-HCV IgG, and serum ALT levels were quantified with colormetric assay (Drew Scientific, Huston, TX). Cut off for normal ALT activity level values was calculated separately for each chimpanzee from 9 or 10 weekly serum specimens obtained before HCV inoculations by adding the mean value to its 3 standard deviations (99.7% confidence interval).

### Frozen liver biopsy samples

Liver needle biopsy samples were obtained before inoculation and every week after inoculation. Samples were frozen in liquid nitrogen immediately after the biopsy and subsequently stored at -80°C until further use. For CH256, 6 liver biopsy samples was obtained before inoculation (−56, −44, −37, −31, −24 and 0 days after inoculation [DAI]) and 13 samples after inoculation (DAIs 4, 12, 32, 40, 53, 75, 82, 95, 103, 124, 145, 152, and 180). For CH6413, 5 liver samples obtained before inoculation (DAIs −44, −37, −31, −16, and 0) and 14 samples after inoculation (DAIs 4, 12, 19, 32, 40, 47, 53, 61, 68, 82, 88, 103, 152, and 180) were used. For CH1541, 5 samples were taken before inoculation (DAIs −53, −24, −16, −7, and 0) and 11 samples after inoculation (DAIs 4, 12, 32, 40, 47, 53, 75, 82, 110, 152, and 173). HCV RNA in snap-frozen liver biopsy specimens was quantified by Taqman real-time PCR using 7900 HT fast real-time PCR system (Applied Biosystems, Foster City, CA). Primers and probe sequences were derived from 5′ non-coding region of the HCV genome (forward primer: 5′-GTCTGCGGAACCGGTGAG-3′) reverse primer: 5′- CGACCCAACRCTACTCGGCTAG-3′, probe: 5′-ACACCGGAATTGCCAGGACGACC-3′).

### Real-time PCR for miR-122

Total RNA from the frozen liver specimens was extracted using the Ribo Pure kit (Ambion, Austin, TX) and 2 pmol of *Caenorhabditis elegans* miR-39 (Qiagen, Valencia, CA) was spiked to total RNA as quality control RNA for real-time PCR. Quality and quantity of the total RNA was analyzed by the 2100 Bioanalyzer (Agilent Technologies, Santa Clara, CA). Total RNA was polyadenylated and reverse transcribed in 10 µl volumes using the miScript reverse transcription (RT) kit (Qiagen, Valencia, CA). All real-time PCR reactions were performed in triplicate on a 384-well 7900 HT fast real-time PCR system (Applied Biosystems, Foster City, CA) including non-template controls. Each real-time PCR reaction was done twice at different times. The reaction mix was incubated for 15 min at 95°C and 10 min at 50°C, then followed by 55 PCR cycles, each cycle comprising 94°C for 15 s, 55°C for 30 s and 70°C for 45 s. Data were analyzed with SDS Software version 2.3 (Applied Biosystems, Foster City, CA) with automatic baseline and threshold settings for cycle threshold (Ct) determination.

For determination of miR-122 levels in serum, total RNA was extracted from 200 µl of each serum sample by TRI-reagent (Life Technologies, Grand Island, NY) according to the manufacturer's recommendations. Prior to RNA extraction, 2 pmol SV-40 miRNA (Qiagen, Valencia, CA) was added to each serum sample for normalization. Then, cDNA synthesized from 1/10 of reverse transcription reaction from serum specimens was used for real-time PCR using miScript SybrGreen PCR kit (Qiagen, Valencia, CA) according to the manufacturer's recommendations.

### Normalization of miR-122 results

The NormFinder program [25] was used to select unbiased endogenous control genes for normalization in both non-infected and HCV-infected liver tissues separately for each chimpanzee. Candidate genes used included six small RNAs [RNU6 (MS00033740), SNORD61 (MS00033705), SNORD68 (MS00033712), SNORD96A (MS00033733), SNORD95 (MS00033726), SNORD72 (MS00033719] and six miRNAs [miR-191 (MS00003682), miR-103a (MS00031241), miR-17 (MS00029274), let-7b (MS00003122), miR-15a (MS00003178), *C. elegans* miR-39 (MS000197890)]. All of these candidate control RNAs have been reported to be expressed in the liver and six miRNAs were used as endogenous control genes in previous studies [21], [26]. Primers for the small RNAs and miRNAs including miR-122 (MS00003416) were obtained from Qiagen. Relative quantification of the miRNA and mRNA expression was calculated using the 2^−ΔΔCt^ method [27]. Greater than 2-fold increases of genomic expressions were considered to be significant. miR-122 values in serum samples were normalized by the levels of added SV40 tracer [28]. miR-122 values in liver tissue and serum samples obtained before HCV inoculations were used as calibrators. Two groups of liver samples were included, those taken before HCV inoculation (n = 6 in CH256, n = 5 in CH6413 and CH1541) and those after HCV inoculation (n = 13 in CH256, n = 14 in CH6413, and n = 11 in CH1541). For each small RNA and miRNA control, the Ct values were calculated separately for each chimpanzee and then the relative quantities (RQ) for each control gene across all samples were calculated from Ct values scaled to a calibrator sample (lowest Ct) by using the equation: RQ = 1/(2^(Ct sample−Ct min)^) [29]. Only the small RNA or miRNAs controls with the lowest intergroup variation and intragroup variation were selected for normalization. Geometric mean Ct values from these selected control RNAs were calculated and then subtracted from the miR-122 Ct (ΔCt miR-122). Liver samples obtained before HCV inoculation were used as calibrator samples, and the arithmetic mean Ct of each calibrator sample (n = 6 in CH256, n = 5 in CH6413 and n = 5 CH1541) was calculated. Fold change of miR-122 expression relative to the endogenous control genes was determined by the 2^−ΔΔCT^ method. The ΔΔCt was obtained by mean Ct of the calibrator samples (Ct miR-122 – Ct endogenous control genes) subtracted from ΔCt miR-122 (Ct miR-122 – Ct endogenous control genes).

### IFN-α and IFN-β gene expression in the liver

cDNA was synthesized with 1 µg of total RNA using a high capacity cDNA reverse transcription kit (Applied Biosystems, Foster City, CA) and used as template for real-time PCR performed by the TaqMan method. The reaction mix was incubated for 5 min at 95°C and 10 min at 50°C, followed by 40 PCR cycles, each cycle comprising 95°C for 15 s and 60°C for 1 min. Glyceraldehyde 3-phosphate dehydrogenase (GAPDH, 4326317E) was used as internal control and a preinoculation sample used as calibrator. Primers and internal probes for IFN-α (Hs00256882_s1) and IFN-β (Hs02621180_s1) were obtained from Applied Biosystems. All of real-time PCR reactions were performed in triplicate on the 7900 HT fast real-time PCR system (Applied Biosystems, Foster City, CA). Transcripts of each gene were calculated based on the 2^−ΔΔCT^ method [27].

### Statistical evaluation

Statistical analyses were carried out using SPSS software 20 (Chicago, IL). Differences were detected using analysis of variance (ANOVA). A positive or negative correlation was indicated by the Spearman correlation coefficient and verified by two-tailed significance test. For all statistical tests, with a *p* value <0.05 considered significant. The correlations were calculated separately for each chimpanzee and also a single analysis was performed with data from all three animals.

## Results

### Selection of the endogenous miRNA for miR-122 expression normalization

To identify the most stable endogenous RNA as normalizers in hepatic miR-122 expression, twelve small RNAs and miRNAs were selected as candidate endogenous control RNAs. Ct values of *C. elegance* miR-39, used as a spike-in RNA control to assess the quality control and confidence of the real-time PCR performance, were similar in all samples from all three chimpanzees included in the study (Table S1). The twelve endogenous control RNAs were expressed in all samples, with Ct values ranging from 22.36 to 36.74 in CH256, 18.08 to 44.88 in CH6413, and 22.24 to 35.42 in CH1541. miR-103 was not used for further calculation in CH6413 due to its low expression (from 32.62 to 44.88, average Ct 41.43) (Table S1). miR-122 Ct values were observed in range from 20.68 to 29.01 for CH256, 23.74 to 29.03 for CH6413, and 22.79 to 27.78 for CH1541, i.e., in range of Ct values of the endogenous controls. The ANOVA-based model to estimate intra- and inter-group variation was used to select endogenous control genes for unbiased normalization across all samples individually in each chimpanzee [25]. It determined a stability value for each endogenous control gene and indicated the single most stable endogenous control for each animal and endogenous control gene pair for which the stability of the latter was greater than that of the single endogenous control. Two Ct values of each endogenous control obtained from two separate reactions were combined and the mean Ct value was used for calculation. The combination of SNORD95 and SNORD72 was selected for two chimpanzees, CH256 and CH6413, and the combination of SNORD95 and miR-191 was selected for CH1541 (Table 1).

**Table 1:** Selection of the most stable endogenous control genes for miR-122 results normalization in HCV-inoculated chimpanzees [25].

	CH256		CH6413		CH1541	
Rank	Gene name	Stability value	Gene name	Stability value	Gene name	Stability value
Best gene combination	SNORD95 and SNORD72	0.245	SNORD95 and SNORD72	0.188	SNORD95 and miR-191	0.112
1	SNORD61	0.315	SNORD96A	0.390	miR-191	0.131
2	SNORD95	0.467	SNORD72	0.412	SNORD95	0.181
3	let-7b	0.467	let-7b	0.414	miR-103	0.206
4	miR-17	0.473	SNORD61	0.417	SNORD72	0.224
5	RNU6	0.607	SNORD95	0.426	let-7b	0.241
6	SNORD72	0.661	RNU6	0.458	SNORD68	0.261
7	miR-15a	0.694	miR-15a	0.550	RNU6	0.273
8	SNORD96A	0.781	miR-191	0.706	SNORD96A	0.292
9	miR-39	0.819			SNORD61	0.383
10	SNORD68	0.832			miR-17	0.443
11					miR-39	0.700

### miR-122 expression and HCV RNA titer in the liver and serum

**Figure 1:**
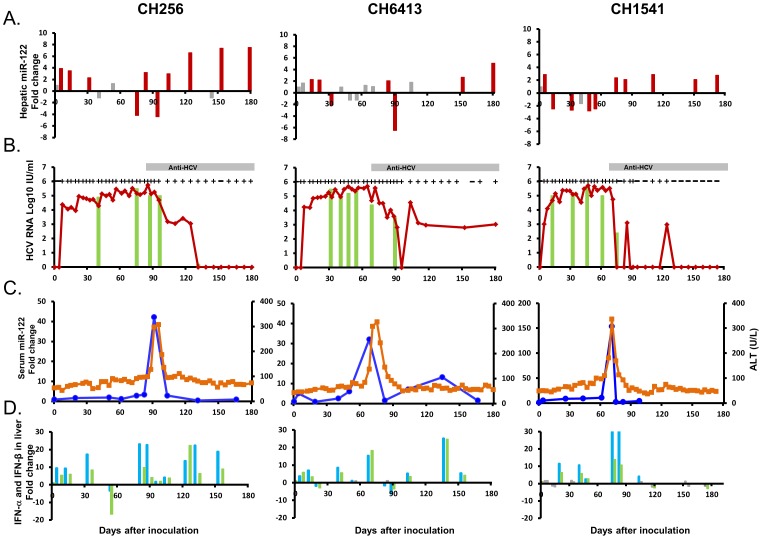
Acute HCV infection in chimpanzees CH256, CH6413, and CH1541. (A) Hepatic miR-122: red bars - up and down-regulated expression-fold change >2.0; gray bars -fold change <2.0. (B) HCV RNA titer in serum and liver: red lines represent serum HCV RNA; green bars represent HCV RNA titer in the liver. “+” denotes presence and “–” absence of HCV RNA in serum (detection limit 600 IU/ml). (C) Serum miR-122 levels -blue line; ALT levels- orange line. (D) IFN-α (blue bar), and IFN-β (green bar) mRNA expression.

Normalized levels of miR-122 in the liver increased from 2.2 to 3.9 times those of pre-inoculation samples: from DAIs 4 to 32 for CH256, DAIs 4 to 19 for CH6413, and on DAI 4 for CH1541 (Fig. 1A). During the peak of HCV replication, miR-122 expression had either decreased or remained unchanged (DAIs 40 to 95 in CH256; DAI 32 to 88 in CH6413; and DAIs 12 to 53 in CH1541). In all three animals, hepatic miR-122 levels were on the rise coinciding with the period when HCV RNA titers were declining (Fig. 1A and B).

**Figure 2:**
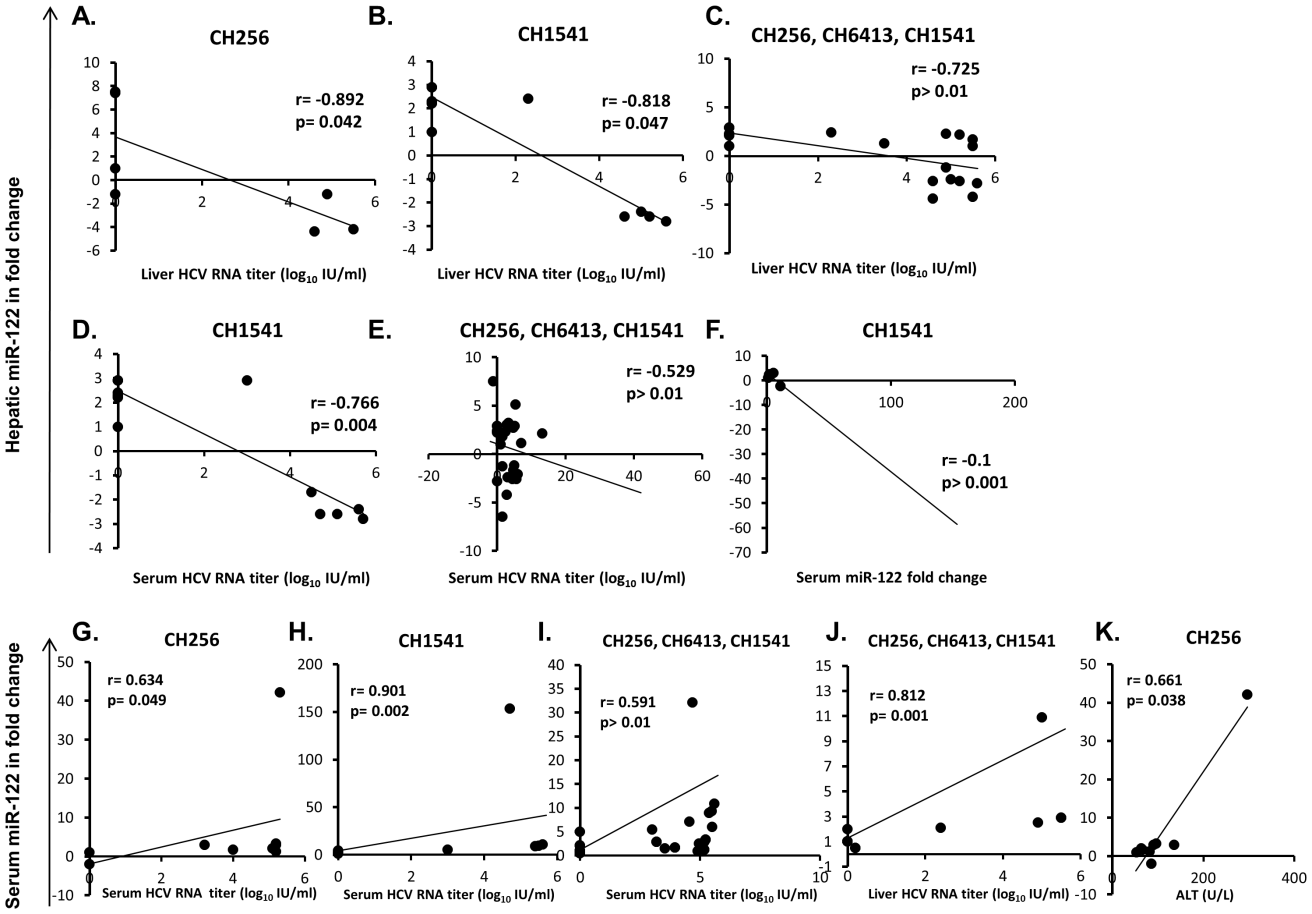
Acute HCV infection in chimpanzees. Hepatic and serum miR-122 statistically significant correlation with liver, serum HCV RNA titers, and ALT activity. Hepatic miR-122 vs. HCV RNA titer in the liver in CH256 (A); in CH1541 (B); in CH256, CH6413, CH1541 (C); vs. HCV RNA titer in serum in CH1541 (D); in CH256, CH6413, CH1541 (E); vs. serum miR-122 in CH1541 (F). Serum miR-122 vs. HCV RNA titer in serum in CH256 (G); in CH1541 (H); in CH256, CH6413, CH1541 (I); vs. HCV RNA titer in the liver in CH256, CH6413, CH1541 (J); vs. ALT activity in CH256 (K).

All three were HCV RNA-positive in serum from DAIs 4 (CH1541) or 7 (CH256 and CH6413) and viral titers were maintained until DAI 71 (CH1541) or DAI 95 (CH256 and CH6413) (Fig. 1B). A similar profile was observed for HCV RNA in the liver; about 2-log decline in liver viral RNA levels was detected at DAI 75 (CH1541), 131 (CH256) or 88 (CH6413) (Fig. 1B). ALT activity was significantly elevated at DAI 71 (CH1541), 95 (CH256) or 75 (CH6413) (Fig. 1C, Table S2). A statistically significant inverse correlation was found between hepatic miR-122 expression and liver HCV RNA titers in CH256 and with both liver and serum HCV RNA titers in CH1541 (Fig. 2A, B, and D, Table S3A). A statistically significant negative correlation between hepatic miR-122 expression and the levels of serum miR-122 was found in CH1541 (Fig. 2F, Table S3A). Serum miR-122 levels were positively correlated with serum HCV RNA titer in CH256 and CH1541 (Fig. 2G and H, Table S3B). When hepatic and serum miR-122 data and all other parameters from all three chimpanzees were included in one analysis, a statistically significant inverse correlation between hepatic miR-122 and HCV RNA level in the liver and serum was observed (Fig. 2C and E, Table S3C). Levels of serum miR-122 were positively correlated with HCV RNA titer in the liver and serum (Fig. 2I and J, Table S3C). Expression profiles of serum miR-122 detected in three chimpanzees were similar to those of ALT activity (Fig. 1C); the correlation between those two measures was statistically significant in CH256 (Fig. 2K, Table S3B).

### Type-1 interferon-induced mRNAs in the liver

**Figure 3:**
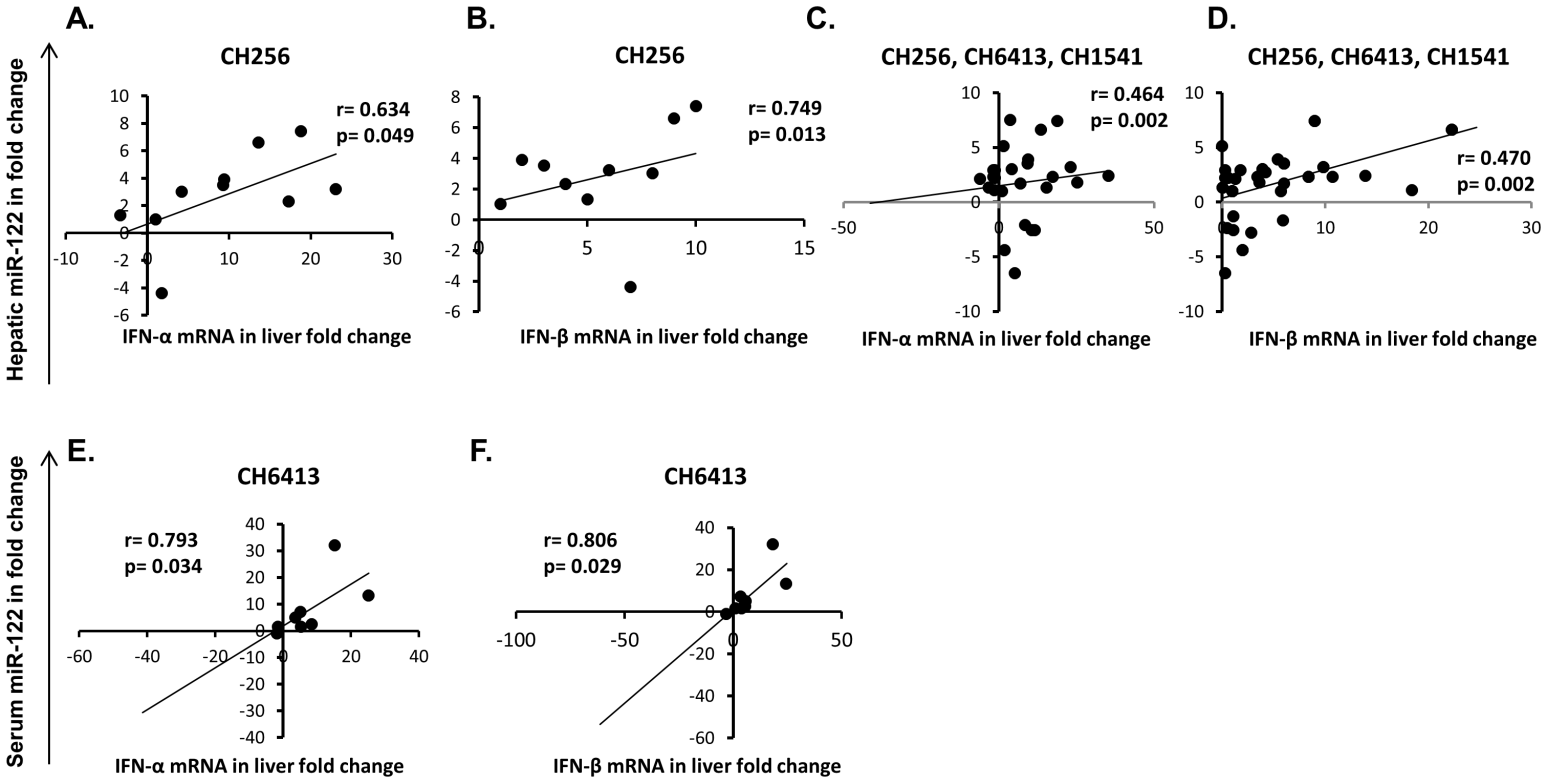
Correlation between hepatic/serum miR-122 expression and hepatic IFN-α/β mRNA liver expression level in chimpanzees with acute HCV infection. Hepatic miR-122 levels vs. hepatic IFN-α mRNA levels in CH256 (A); vs. IFN-β mRNA levels in CH256 (B); vs. hepatic IFN-α mRNA levels in CH256, CH6413, CH1541 (C); vs. IFN-β mRNA levels in CH256, CH6413, CH1541 (D). Serum miR-122 levels vs. hepatic IFN-α mRNA levels (E), and IFN-β mRNA levels (F) in CH6413.

High levels of hepatic IFN-α and IFN-β mRNAs were detected during the period when levels of hepatic miR-122 expression were in decline (Fig. 1D, Table S2). Hepatic IFN-α and IFN-β mRNA expressions were correlated with hepatic miR-122 levels in CH256 (Fig. 3A and B, Table S3A). In CH6413, the levels of IFN-α and IFN-β mRNAs were correlated with serum miR-122 levels (Fig. 3E, F, and Table S3B). When all parameters from all three chimpanzees were included in one analysis, the moderate, but statistically significant positive correlation between hepatic IFN-α and IFN-β mRNA and hepatic miR-122 levels was observed (Fig. 3C and D, Table S3C).

## Discussion

The kinetics of miR-122 in the liver and serum during the acute phase of HCV infection were determined in chimpanzees inoculated with HCV genotype 1a. Levels of hepatic miR-122 genomic expression were evaluated by quantitative real-time RT-PCR (qRT-PCR) with the values stringently normalized using selected host genes to correct for biological and experimental bias and for inter-assay variations in each of these chimpanzee included in the study [25], [29]. The combination of SNORD95/SNORD72 was selected for CH256 and CH6413, and SNORD95 and miR-191 for CH1541 as the most stable endogenous control genes (Table 1). The RNU6B (U6) gene used in other studies for evaluation of miR-122 expression [21], [30], [31] has proven to be an unreliable normalizer [26], [32], [33].

Up-regulated expression of miR-122 in the liver was observed during the first four weeks after HCV inoculation in all three chimpanzees, at the beginning of HCV viremia and before any evidence of liver injury as measured by serum ALT elevation (Fig. 1A, B, C, and Table S2). Temporal concordance between increasing levels of miR-122 and rapidly increasing HCV RNA titer in the liver and blood has been consistent with data obtained *in vitro* which showed augmentation of HCV RNA accumulation in Huh7 cells consequential to expression of miR-122 [3], [4]. The kinetics of miR-122 expression during the acute phase of HCV infection further may validate positive impact of miR-122 on viral abundance and its role in the enhancement of HCV replication and stability. The positive influence of miR-122 on HCV replication has also been suggested by a study of chronically HCV-infected chimpanzees who were treated with antisense miR-122 and then achieved long-lasting suppression of HCV viremia and chronic hepatitis C patients treated with DNA antisense nucleotide that sequesters mature miR-122 [18], [19].

During the course of acute HCV infection in our chimpanzees, when hepatic and serum HCV RNA titers had increased 4 to 5 logs, a trend was observed for hepatic miR-122 values either to have fallen or remain unchanged; for CH1541, the phase of decline in hepatic levels of miR-122 preceded quite distinctly the appearance of miR-122 in serum. The inverse relationship between hepatic miR-122 and hepatic HCV RNA titers was statistically significant in CH256 and CH1541, and was even more statistically apparent when data from all three animals were evaluated in a single analysis (Fig.2 and Table S3C). In addition, the statistically significant negative correlation between hepatic miR-122 expression and serum level of HCV RNA was observed in CH1541. It is conceivable that the decrease of hepatic miR-122 levels could be due to release of proteins from degraded hepatocytes upon injury as such decrease also coincided with the rise of ALT activity. An inverse correlation between hepatic miR-122 and HCV RNA level in serum has previously been reported in patients with chronic HCV infection [31]. Although a previous *in vitro* study documented suppression of miR-122 expression by IFN-β [11], and interferon repression of miR-122 in the liver was suggested from clinical observations of interferon-treated patients with chronic hepatitis C [21], the association between decreased miR-122 and increased genomic expression of IFN-α and IFN-β in the studied chimpanzees was not found. To the contrary, there was a statistically significant positive correlation between hepatic miR-122 and type 1 interferon in CH256 and when all chimpanzee data were tested in a single analysis (Fig. 3 and Table S3A, C).

Towards the later phase of the acute HCV infection, hepatic levels of miR-122 in the chimpanzees were observed to have risen again, corresponding to the phase of declining HCV RNA titers in the liver and clearance of HCV RNA from serum (Fig. 1A). The mechanism underlying the miR-122 kinetics in this phase of acute infection is unclear. It is also unknown for how long this upsurge of miR-122 expression in the liver could have been maintained, or whether these rises predict the development of chronic HCV infection or resolution of acute infection, as the chimpanzees in our study were not tested beyond 180 days after HCV inoculation.

In summary, a tri-phasic change in miR-122 levels in the liver was observed in CH256 and less prominently in two other chimpanzees during acute HCV infection. During the initial phase of the infection, rising hepatic miR-122 levels and HCV RNA in liver and serum likely reflects the phase of infection when miR-122 is participating in the enhancement of HCV replication, as reported from *in vitro* studies [4], [7], [13], [15]–[17]. The second phase, when miR-122 levels in the liver are in a decline, may correspond to the phase of destruction of HCV-infected hepatocytes and release or transport of miR-122 into the plasma. The mechanisms underlying the third phase, of a renewed upsurge in miR-122 levels in the liver, remain unclear, although it may be related to the restoration of morphologic integrity of the liver during the HCV clearance and liver lesions healing. Kinetics of changes in miR-122 expression in the liver observed *in vivo* document the complexities of the virus-host interaction during the acute phase of HCV infection.

## Supporting Information

Table S1 (XLSX)

Table S2 (DOCX)

Table S3 (DOCX)
