# Supplementary material for: Kinetics of miR-122 Expression in the Liver during Acute HCV Infection
Source: PLoS One. 2013 Oct 4;8(10):e76501. doi: 10.1371/journal.pone.0076501 (PMC3790687; doi:10.1371/journal.pone.0076501)
Supplement: Table S2 — (DOCX) [file pone.0076501.s002.docx]

Supplemental Table S2. Acute HCV infection in chimpanzees. HCV RNA titer in serum and liver, ALT activity and fold-changes in hepatic and serum miR-122, and IFN-α, IFN-β, and CAT-1 gene expression in sequential liver biopsy and serum samples.

| **Chimp** | **DAI** | **miR-122** | | **HCV RNA titer Log_10_** | | **ALT (U/L) (c/o=61)** | **hepatic mRNA expression**  **[fold change]** | |
| --- | --- | --- | --- | --- | --- | --- | --- | --- |
|  |  | **liver**  **[fold change]** | **Serum**  **[fold change]** | **serum (IU/ml)^#^** | **liver (IU/ml)^%^** |  | **IFN-α** | **IFN-β** |
| **CH256** | -31 | 1 | 1 | 0 |  | 55 |  |  |
|  | -24 | 1 | 1 | 0 |  | 55 |  |  |
| **7 yr,** | -16 | 1 | 1 | 0 |  | 46 |  |  |
| **male,** | 0 | 1 | 1 | 0 | 0 | 54 | 1 | 1 |
| **34 kg** | 4 | 3.9 |  | 0 (-) |  | 56 | 9.4 | 5.4 |
|  | 12 | 3.5 |  | 4.1 |  | 60 | 9.3 | 6 |
|  | 19 |  | 1.7 | 4 |  | 67 |  |  |
|  | 32 | 2.3 |  | 4.7 |  | 51 | 17.3 | 8.4 |
|  | 40 | -1.2 |  | 4.3 | 4.9 | 55 |  |  |
|  | 50 |  | 2 | 5.1 |  | 64 |  |  |
|  | 53 | 1.3 |  | 4.7 |  | 91 | -3.3 | -16.7 |
|  | 61 |  | 1.2 | 5.2 |  | 83 |  |  |
|  | 75 | -4.2 | 2.9 | 5.2 | 5.5 | 91 |  |  |
|  | 82 | 3.2 | 3.3 | 5.2 |  | 97 | 23.1 | 9.8 |
|  | 88 |  |  | 5.1 | 5 | 128 | 22.7 | 4.2 |
|  | 91 |  | 42.1 | 5.3 |  | 297 |  |  |
|  | 95 | -4.4 |  | 4.7 | 4.6 | 107 | 1.8 | 2 |
|  | 103 | 3 | 2.9 | 3.2 |  | 136 | 4.2 | 3.9 |
|  | 124 | 6.6 |  | 3 |  | 87 | 13.6 | 22..3 |
|  | 131 |  | -2 | 0 (+) | 0.2 | 86 | 22.4 | 6.4 |
|  | 145 | -1.2 |  | 0 (+) |  | 73 |  |  |
|  | 152 | 7.4 |  | 0 (-) |  | 57 | 18.8 | 9 |
|  | 180 | 7.5 |  | 0 (+) |  | 74 |  |  |
|  |  |  |  |  |  | (c/o=58) |  |  |
| **CH6413** | -56 | 1 | 1 | 0 |  | 42 |  |  |
|  | -37 | 1 | 1 | 0 |  | 37 |  |  |
| **6 yr,** | -31 | 1 | 1 | 0 |  | 45 |  |  |
| **female,** | 0 | 1 | 1 | 0 | 0 | 43 | 1 | 1 |
| **32 kg** | 4 | 1.7 | 5 | 0 (+) |  | 46 | 3.7 | 6 |
|  | 12 | 2.3 |  | 4.2 |  | 49 | 7 | 3.4 |
|  | 19 | 2.2 | -1.1 | 4.9 |  | 56 | -1.8 | -3 |
|  | 32 | -2.1 |  | 4.9 | 5.5 | 52 |  |  |
|  | 40 | 1 | 2.5 | 5 | 4.9 | 67 | 8.5 | 5.7 |
|  | 47 | -1.3 |  | 5.7 | 5.2 | 64 |  |  |
|  | 50 |  | 6 | 5.5 |  | 65 |  |  |
|  | 53 | -1.3 |  | 5.4 | 5.5 | 83 | 1.2 | 1.1 |
|  | 61 | 1.3 |  | 5.6 |  | 69 |  |  |
|  | 68 | 1.1 | 32.1 | 4.7 | 4.4 | 137 | 15.3 | 18.4 |
|  | 82 | 2.1 | 1.5 | 3.5 |  | 145 | -1.5 | 1.3 |
|  | 88 | -6.5 |  | 3.6 | 3.5 | 92 | -6.1 | -3.5 |
|  | 103 | 1.8 | 7.1 | 4.6 |  | 52 | 5.2 | 3.6 |
|  | 138 |  | 13.2 | 0 (+) | 0 | 60 | 25.2 | 24.7 |
|  | 152 | 2.7 | 1.5 | 2.8 |  | 73 | 5.3 | 4.2 |
|  | 166 |  | 1.5 | 0 (+) |  | 56 |  |  |
|  | 180 | 5.1 |  | 3 |  | 55 | -41.4 | -61.5 |
|  |  |  |  |  |  | (c/o=60) |  |  |
| **CH1541** | -24 | 1 | 1 | 0 |  | 40 |  |  |
|  | 0 | 1 | 1 | 0 | 0 | 49 | 1 | 1 |
| **12 yr** | 4 | 2.9 | 5.4 | 3 |  | 50 | 1.5 | 1.8 |
| **female,** | 12 | -2.5 |  | 4.7 | 4.6 | 45 | -1.2 | -1.9 |
| **56 kg** | 19 |  |  | 4.6 |  | 56 | 11.5 | 6.4 |
|  | 26 |  | 8.9 | 5.4 |  | 66 |  |  |
|  | 32 | -2.7 |  | 5.1 | 5.2 | 56 | 1.7 | 1.1 |
|  | 40 | -1.7 |  | 4.5 |  | 79 | 10.6 | 5.9 |
|  | 43 |  | 9.3 | 5.5 |  | 82 |  |  |
|  | 47 | -2.8 |  | 5.7 | 5.6 | 63 | 2.6 | 2.8 |
|  | 53 | -2.5 | 10.9 | 5.6 | 5 | 78 |  |  |
|  | 71 |  | 153.4 | 4.7 |  | 336 |  |  |
|  | 75 | 2.4 | 2.1 | 0 (+) | 2.3 | 169 | 32.9 | 13.9 |
|  | 82 | 2.1 | 2 | 0 (+) | 0 | 93 | 35.4 | 10.7 |
|  | 85 |  |  | 3.1 |  | 71 |  |  |
|  | 95 |  | 4.2 | 0 (-) |  | 48 |  |  |
|  | 103 |  |  | 0 (-) |  | 41 | 4.1 | 1.3 |
|  | 110 | 2.9 |  | 0 (+) |  | 43 |  |  |
|  | 117 |  |  | 0 (+) |  | 53 | -1.8 | -2.5 |
|  | 134 |  |  | 0 (-) |  | 55 |  |  |
|  | 152 | 2.2 |  | 0 (-) |  | 51 | 1.2 | -1.6 |
|  | 173 | 2.8 |  | 0 (-) | NEG | 47 | -1.2 | -2.9 |

DAI, days after inoculation; Fc, fold change of expression compared to uninfected samples; c/o, cut-off value of ALT activity. #, HCV RNA serum titer was measured using Cobas Amplicor HCV v. 2.0 (Roche Diagnostic Systems, Branchburg, NJ). %, HCV RNA titer in the liver was quantified by in-house Taqman real-time PCR.
