# Supplementary material for: Kinetics of miR-122 Expression in the Liver during Acute HCV Infection
Source: PLoS One. 2013 Oct 4;8(10):e76501. doi: 10.1371/journal.pone.0076501 (PMC3790687; doi:10.1371/journal.pone.0076501)
Supplement: Table S3 — (DOCX) [file pone.0076501.s003.docx]

Supplemental Table S3. Correlation between miR122 in liver and serum, hepatic IFN-α and IFN-β, and ALT profiles during 180 days of acute HCV infection in chimpanzees (Spearman correlation test)

**A.**

|  | **Hepatic miR-122**  **vs.** | |  |  |  |  |
| --- | --- | --- | --- | --- | --- | --- |
| **Chimp** | **HCV RNA in liver** | **HCV RNA**  **in serum** | **ALT**  **in serum** | **IFN-α**  **in liver** | **IFN-β**  **in liver** | **miR-122**  **in serum** |
|  |  |  |  |  |  |  |
| **CH256** | r= -0.892 | r= -0.492 | r= -0.237 | r= 0.634 | r= 0.749 | r= 0.272 |
|  | p= 0.042 | p= 0.074 | p= 0.415 | p= 0.049 | p= 0.013 | p= 0.728 |
|  |  |  |  |  |  |  |
| **CH6413** | r= -0.385 | r= -0.474 | r= -0.208 | r= 0.007 | r= -0.026 | r= 0.000 |
|  | p= 0.394 | p= 0.074 | p= 0.457 | p= 0.982 | p= 0.936 | p= 1.0 |
|  |  |  |  |  |  |  |
| **CH1541** | r= -0.818 | r= -0.766 | r= -0.326 | r= -0.103 | r= -0.006 | r= -1.0 |
|  | p= 0.047 | p= 0.004 | p= 0.302 | p= 0.778 | p= 0.986 | p> 0.01 |
|  |  |  |  |  |  |  |

**B.**

|  | **Serum miR-122**  **vs.** | |  |  |  |
| --- | --- | --- | --- | --- | --- |
| **Chimp** | **HCV RNA**  **in liver** | **HCV RNA**  **in serum** | **ALT**  **in serum** | **IFN-α**  **in liver** | **IFN-β**  **in liver** |
|  |  |  |  |  |  |
| **CH256** | r= 0.866 | r= 0.634 | r= 0.661 | r= 0.211 | r= 0.211 |
|  | p= 0.333 | p= 0.049 | p= 0.038 | p= 0.789 | p= 0.789 |
|  |  |  |  |  |  |
| **CH6413** | r= 0.500 | r= 0.397 | r= 0.306 | r= 0.793 | r= 0.806 |
|  | p= 0.667 | p= 0.331 | p= 0.391 | p= 0.034 | p= 0.029 |
|  |  |  |  |  |  |
| **CH1541** | r= -0.833 | r= 0.912 | r= 0.455 | r= 0.316 | r= 0.316 |
|  | p= 0.167 | p= 0.002 | p= 0.230 | p= 0.684 | p= 0.684 |
|  |  |  |  |  |  |

| **C.** | **Hepatic miR-122**  **vs.** | |  |  |  |  |
| --- | --- | --- | --- | --- | --- | --- |
| **Chimp** | **HCV RNA**  **in liver** | **HCV RNA**  **in serum** | **ALT**  **in serum** | **IFN-α**  **in liver** | **IFN-β**  **in liver** | **miR-122**  **in serum** |
|  |  |  |  |  |  |  |
| **CH256, CH6413, CH1541** | r= -0.725 | r= -0.529 | r=-0.122 | r= 0.464 | r= 0.470 | r= 0.320 |
|  | p> 0.01 | p> 0.01 | p= 0.376 | p= 0.002 | p= 0.002 | p= 0.182 |
|  |  |  |  |  |  |  |

|  | **Serum miR-122**  **vs.** | |  |  | | |  | |  | |
| --- | --- | --- | --- | --- | --- | --- | --- | --- | --- | --- |
| **Chimp** | **HCV RNA**  **in liver** | **HCV RNA**  **in serum** | **ALT**  **in serum** | | | **IFN-α**  **in liver** | | **IFN-β**  **in liver** | |  |
|  |  |  |  | |  | | |  | |  |
| **CH256, CH6413, CH1541** | r= 0.812 | r= 0.591 | r= 0.292 | | r= 0.232 | | | r= 0.396 | |  |
|  | p= 0.001 | p> 0.01 | p= 0.071 | | p= 0.340 | | | p= 0.093 | |  |
|  |  |  |  | |  | | |  | |  |

No. of samples used: CH256: liver n=13, serum n=13; CH6413: liver n=14, serum n=13; and CH1541: liver n=11, serum n=11.
